# The Multifunctional Long-Distance Movement Protein of *Pea Enation Mosaic Virus 2* Protects Viral and Host Transcripts from Nonsense-Mediated Decay

**DOI:** 10.1128/mBio.00204-20

**Published:** 2020-03-10

**Authors:** Jared P. May, Philip Z. Johnson, Muhammad Ilyas, Feng Gao, Anne E. Simon

**Affiliations:** a Department of Cell Biology and Molecular Genetics, University of Maryland—College Park, College Park, Maryland, USA; Icahn School of Medicine at Mount Sinai

**Keywords:** NMD, RNA stability, virology, movement protein, nonsense-mediated decay, plant viruses, virus-host interactions

## Abstract

The nonsense-mediated decay (NMD) pathway presents a challenge for RNA viruses with termination codons that precede extended 3′ untranslated regions (UTRs). The umbravirus *Pea enation mosaic virus 2* (PEMV2) is a nonsegmented, positive-sense RNA virus with an unusually long 3′ UTR that is susceptible to NMD. To establish a systemic infection, the PEMV2 long-distance movement protein p26 was previously shown to both stabilize viral RNAs and bind them for transport through the plant’s vascular system. The current study demonstrated that p26 protects both viral and nonviral messenger RNAs from NMD. Although p26 localizes to both the cytoplasm and nucleolus, p26 exerts its anti-NMD effects exclusively in the cytoplasm independently of long-distance movement. Using a transcriptome-wide approach in the model plant Nicotiana benthamiana, p26 protected a subset of cellular NMD target transcripts, particularly those containing long, structured, GC-rich 3′ UTRs. Furthermore, transcriptome sequencing (RNA-seq) revealed that the NMD pathway is highly dysfunctional during PEMV2 infection, with 1,820 (48%) of NMD targets increasing in abundance. Widespread changes in the host transcriptome are common during plant RNA virus infections, and these results suggest that, in at least some instances, virus-mediated NMD inhibition may be a major contributing factor.

## Abstract (precis)

Nonsense-mediated decay (NMD) represents an RNA regulatory pathway that degrades both natural and faulty messenger RNAs with long 3′ untranslated regions. NMD targets diverse families of RNA viruses, requiring that viruses counteract the NMD pathway for successful amplification in host cells. A protein required for long-distance movement of *Pea enation mosaic virus 2* (PEMV2) is shown to also protect both viral and host mRNAs from NMD. RNA-seq analyses of the Nicotiana benthamiana transcriptome revealed that PEMV2 infection significantly impairs the host NMD pathway. RNA viruses routinely induce large-scale changes in host gene expression, and, like PEMV2, may use NMD inhibition to alter the host transcriptome in an effort to increase virus amplification.

## INTRODUCTION

Eukaryotic cells have multiple pathways that degrade dysfunctional mRNAs, including no-go decay, nonstop decay, and nonsense-mediated decay (NMD). NMD safeguards cells from expressing truncated proteins from altered transcripts bearing a premature termination codon (PTC), which can have substantive deleterious effects on cellular functions (1). In addition, NMD controls the stability of 5% to 10% of unaltered mRNAs, contributing to regulation of gene expression (2).

NMD factors include “up-frameshift” proteins (UPF1, UPF2, UPF3a, and UPF3b), with UPF1 being the key effector protein (3). Although NMD is regarded as a quality control pathway for pre-mRNA splicing, NMD can occur either dependently or independently of exon junction complexes (EJC), which are deposited ∼20 nucleotides (nt) upstream of exon-exon junctions during splicing (4). EJC-dependent NMD occurs when a ribosome terminates at a PTC with a downstream EJC (a hallmark of incorrect splicing). UPF2 and UPF3 are recruited to UPF1 via the EJC, leading to phosphorylation of UPF1 by SMG1 (2) and triggering endonucleolytic or exonucleolytic cleavage, depending on the eukaryotic system (5, 6). In plants, degradation of NMD-targeted transcripts can take place in subcellular processing bodies (P-bodies), where the UPF1-RNA complex is recruited by SMG7 (6). P-bodies are enriched in NMD factors (7) and have previously been shown to accumulate aberrant mRNAs in yeast (8).

EJC-independent NMD occurs when UPF1 bound to the 3′ untranslated region (UTR) interacts with eukaryotic release factor 3 (eRF3), which is associated with a terminating ribosome, leading to phosphorylation of UPF1 (2). While UPF1 can bind to any accessible RNA, binding is enriched in transcripts that are GC rich (>15% increased GC content compared to average) (9, 10), as well as in transcripts with long 3′ UTRs (>1.5-fold-greater length than average) (11, 12).

Positive-sense RNA viral genomes are highly susceptible to host RNA degradation pathways, including NMD. The genomic organization of many RNA viruses results in extensive 3′ UTRs, since translation of the genomic RNA (gRNA) 5′-proximal open reading frame (ORF) often terminates upstream of ORFs that are expressed from subgenomic RNAs (sgRNAs). UPF1 overexpression is detrimental to RNA viruses (13), and diverse viruses benefit from UPF1 knockdown (14–16). Although unspliced RNA virus transcripts are targeted by NMD in an EJC-independent manner (17), EJC components (MAGOH, PYM1, and RBM8A) were recently found to have antiviral activity against flaviviruses *West Nile virus* and *Zika virus* (ZIKV) (15).

Evidence is emerging that indicates that viruses predisposed to NMD targeting have evolved different strategies to circumvent NMD that include both *cis*-acting RNA elements and *trans*-acting proteins (18, 19). For example, a pyrimidine-rich, 400-nt sequence immediately following the *gag* stop codon in *Rous sarcoma virus* confers NMD resistance to the unspliced viral RNA by recruiting polypyrimidine tract binding protein 1 (PTBP1), which prevents UPF1 from binding (20, 21). In contrast, *Turnip crinkle virus* (TCV) contains an ∼50-nt unstructured region (USR) at the start of its 3′ UTR that can confer NMD resistance to sensitive transcripts (22). Introducing stable secondary structure into the TCV USR without changing its sequence abolished NMD protection, whereas significantly changing the sequence while maintaining the lack of structure maintained protection, suggesting that highly unpaired regions of RNA are inherently NMD resistant when positioned immediately downstream of a termination codon (22). Ribosome recoding (frameshifting and readthrough) sites, which allow translation to continue past termination codons, can also protect NMD-sensitive RNAs (22–24). These infrequent recoding events are proposed to prevent NMD when the continuing ribosome displaces UPF1.

Virus-encoded proteins have also been implicated in conferring protection to NMD-sensitive gRNAs, as well as host mRNAs, by interfering with critical factors in the NMD pathway. For example, the human T-cell lymphotropic virus type I (HTLV-1) Tax protein binds to the helicase domain of UPF1, reducing its ability to bind RNA and promote NMD (25). HTLV-1 Tax expression also alters the morphology of P-bodies, representing a major site of NMD decay (26). In addition, the HTLV-1 Rex protein, which promotes nuclear export of unspliced and singly spliced viral RNAs, protects NMD-sensitive viral and host mRNAs by disrupting NMD through an unknown mechanism (27). ZIKV capsid protein targets nuclear UPF1 for proteasomal degradation, causing a global inhibition of NMD (28). The viral core protein of *Hepatitis C virus* (HCV) disrupts the interaction between host protein WIBG and EJC components, attenuating NMD (29). Finally, the *Cauliflower mosaic virus* (CaMV) transactivator (TAV) protein interferes with the VARICOSE decapping complex, which is likely required late in the plant NMD pathway (30).

**FIG 1:**
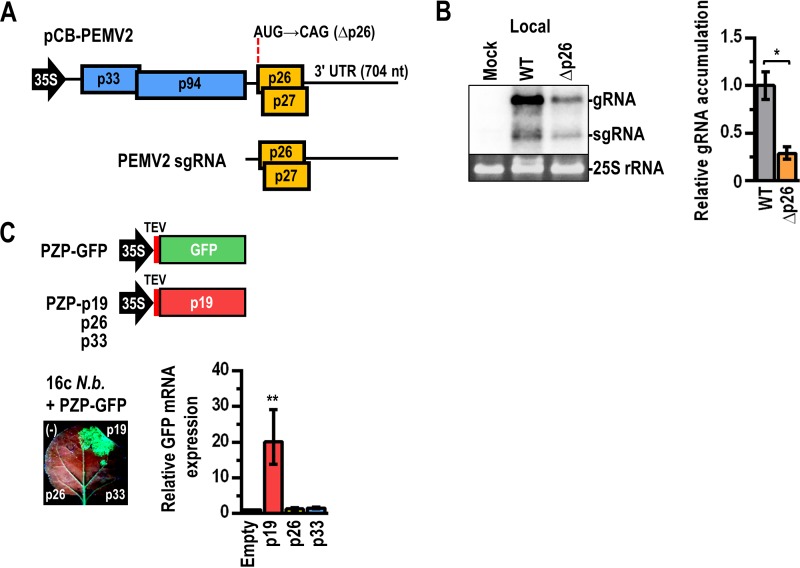
PEMV2 p26 is required for efficient virus accumulation in local leaves and lacks RNA silencing suppressor activity. (A) Genomic organization of PEMV2. The replicase-associated protein (p33) and RNA-dependent RNA polymerase (p94) (generated by ribosome frameshifting prior to the p33 termination codon) are translated from the gRNA, whereas the cell-to-cell and long-distance MPs (p27 and p26, respectively) are translated from a sgRNA that contains a 704-nt 3′ UTR. Full-length transcripts of PEMV2 synthesized from the *Cauliflower mosaic virus* (CaMV) 35S promoter following agroinfiltration of N. benthamiana are capable of replication and movement independently of a helper virus (74); however, the enamovirus *Pea enation mosaic virus 1* (PEMV1) is required for encapsidation and for transmission from plant to plant (75, 76). (B) PEMV2 containing a mutation in the p26 start codon (PEMV2Δp26) was subjected to agroinfiltration along with the p14 silencing suppressor to determine effects on virus accumulation in local (infiltrated) leaves. Viral RNA levels were examined by both Northern blotting (left) and RT-qPCR (right). Ethidium bromide-stained 25S rRNA was used as a loading control in the Northern blot. WT, wild-type PEMV2; Δp26, PEMV2Δp26. Error bars denote standard errors of results from three paired biological replicates *, *P < *0.05 (paired *t* test). (C) PEMV2 p26 was tested for RNA silencing suppressor activity using N. benthamiana line 16c (16c *N.b*.), which constitutively expresses GFP. (Top) PZP-p19, containing the p19 RNA silencing suppressor from TBSV expressed from a *Tobacco etch virus* (TEV) translation enhancer, or PZP with p26 or the PEMV2 p33 replication-required protein was subjected to agroinfiltration along with PZP-GFP. The additional expression of GFP from PZP-GFP results in the silencing of all GFP expression. (Lower left) GFP fluorescence at 2 dpi. Note that leaves naturally autofluoresce red. The presence of a silencing suppressor (such as p19) allows the normally suppressed GFP to become visible. (-), only PZP-GFP was subjected to infiltration. (Lower right)Total RNA was isolated from excised spots, and GFP mRNA levels were determined by RT-qPCR, with the value representing infiltration of an empty vector set at 1.0. Error bars denote standard errors of results from 4 biological replicates. **, *P < *0.01 (one-way analysis of variance [ANOVA] with multiple-comparison test versus empty vector).

Virus-encoded proteins that protect the gRNA or sgRNA from NMD by binding to the viral RNAs have not been reported. However, gRNAs can be stabilized by plant virus-encoded movement proteins (MPs), which bind to viral and other RNAs without known specificity and traffic them in a cell-to-cell manner through plasmodesmata and/or over long distances through the vascular system (31, 32). Umbravirus *Pea enation mosaic virus 2* (PEMV2) (family *Tombusviridae*) has a positive-sense, single-stranded gRNA (4.2 kb) that is targeted by NMD (22) and that encodes both cell-to-cell and long-distance MPs (p27 and p26, respectively) (Fig. 1A). The best-studied umbravirus MP is the p26 orthologue from *Groundnut rosette virus* (GRV) known as plasmid ORF3 (pORF3). pORF3 localizes to the nucleolus and interacts with nuclear protein fibrillarin for long-distance (systemic) movement of GRV gRNA (32–35). pORF3 also binds nucleic acids nonspecifically (31), and both pORF3 and PEMV2 p26 were previously shown to be able to increase the stability of *Tobacco mosaic virus* (TMV) gRNA (36). The stabilization activity of pORF3 was posited to result from formation of dense ribonucleoprotein (RNP) particles consisting of pORF3 oligomers, fibrillarin, and viral RNA (31, 34, 35).

We now report that a transcriptome-wide analysis suggests that PEMV2 p26 assists in protecting PEMV2 gRNA and NMD-sensitive host mRNAs bearing long, GC-rich 3′ UTRs from NMD. Since nearly 50% of all natural NMD targets increase in abundance during PEMV2 infection, the virus likely severely impairs the NMD pathway. This impairment likely accounts for a major portion of the substantial transcriptome differences evident during PEMV2 infection, which are also a hallmark of other virus-infected plants.

## RESULTS

### PEMV2 p26 is required for efficient virus accumulation irrespective of long-distance trafficking.

PEMV2 p26 and p27 MPs are expressed from overlapping ORFs on the PEMV2 sgRNA (Fig. 1A) (37). Elimination of p27 expression had no effect on virus accumulation in *Arabidopsis* protoplasts in a previous study (38). In contrast, mutating the p26 start codon (PEMV2Δp26) resulted in a 4-fold decrease in viral RNA levels, suggesting that the p26 long-distance MP has an additional function that enhances accumulation of PEMV2 gRNA in single cells (38). To determine if similar effects would be apparent *in planta*, wild-type (WT) PEMV2 or PEMV2Δp26 was subjected to agroinfiltration along with the p14 RNA silencing suppressor from *Pothos latent virus* (PLV) (39) into leaves of young Nicotiana benthamiana plants, which are widely used as laboratory hosts for a large number of plant viruses. p14 was included for all agroinfiltrations to increase transient gene expression and also to serve as an internal control for reverse transcription-quantitative PCR (RT-qPCR). Since agroinfiltration leads to expression of viral gRNA in nearly every cell within the infiltrated region, assaying gRNA accumulation in infiltrated (local) leaves reflects the efficiency of virus replication/stability independently of virus movement. At 3 days postinfiltration (dpi), PEMV2Δp26 gRNA accumulation in local leaves (i.e., infiltrated leaves) was reduced 4-fold compared with WT PEMV2 (Fig. 1B), similarly to what was previously observed using *Arabidopsis* protoplasts (38) and further supporting the idea of a role for p26 in promoting genome stability independently of long-distance movement.

To determine if p26, by serving as an RNA silencing suppressor, is required for efficient viral RNA levels, we used transgenic N. benthamiana 16c (40) endogenously expressing green fluorescent protein (GFP) (Fig. 1C). When additional GFP is overexpressed by agroinfiltration of plasmid PZP-GFP, both endogenous and infiltrated GFP levels are silenced, substantially reducing GFP fluorescence (40). However, when an RNA silencing suppressor is infiltrated, a patch of strong GFP fluorescence is observed, as the GFP mRNA is no longer silenced. Indeed, coinfiltration of PZP-GFP with the p19 RNA silencing suppressor from *Tomato bushy stunt virus* (TBSV) (41) reversed the silencing and resulted in strong GFP expression (Fig. 1C). However, coinfiltration of PZP-GFP with p26 or PEMV2 replicase-associated protein p33 had no effect on GFP silencing, demonstrating that both p26 and p33 were shown to lack detectable RNA silencing suppressor activity by this assay. Although PEMV2 lacks an RNA silencing suppressor, *Pea enation mosaic virus 1*, an enamovirus and helper virus for PEMV2, possesses an RNA silencing suppressor that likely confers protection to the PEMV2 genome during natural coinfections (42).

### p26 protects both viral and nonviral 3′ UTRs against NMD targeting.

**FIG 2:**
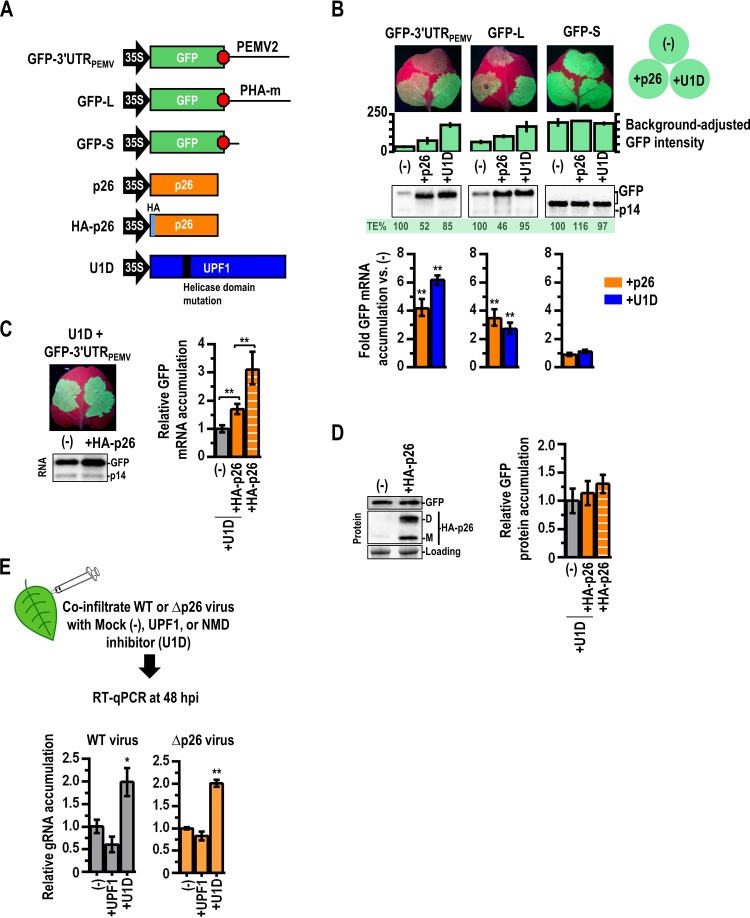
p26 protects NMD-sensitive reporters from NMD. (A) Constructs used in this study. The GFP reporters used were previously described (22). (B) GFP reporters were subjected to agroinfiltration along with p14 and Mock (-), p26, or U1D. (Top) At 48 h postinfection (hpi), infiltrated leaves were examined and GFP intensity was measured using ImageJ. Error bars denote standard deviations of results from at least 5 leaves. (Bottom) Total RNA was extracted from “spots” for Northern blotting (middle) and quantified by RT-qPCR using p14 as a reference. Error bars denote standard errors of results from 3 biological replicates (**, *P < *0.01 [unpaired *t* test]). Translational efficiencies (TE%) were calculated by dividing the mean GFP intensity by the mean mRNA accumulation under each set of conditions, with Mock (-) set at 100%. (C) Effect of p26 expression on GFP-3′UTR_PEMV_ transcript levels in the absence of NMD. GFP-3′UTR_PEMV_ was coinfiltrated with p14 and U1D and either Mock (-) or HA-tagged p26 (HA-p26). At 48 hpi, GFP fluorescence was visualized (left), and GFP mRNA levels were determined by Northern blotting (center) and RT-qPCR (right). Error bars denote standard errors. Paired *t* tests from 5 leaves were used for “+U1D” (**, *P < *0.01). Data representing GFP mRNA levels in the presence of NMD are included as a reference (stripes). An unpaired *t* test was used to compare +HA-p26 samples in the presence or absence of U1D. **, *P < *0.01. (D) (Left) GFP protein levels in the presence or absence of HA-p26 and in the absence of NMD were determined by Western blotting using an antibody against the HA tag. p26 expression resulted in the formation of higher-molecular-weight dimers (“D”) in addition to monomers (“M”), similar to what was observed for GRV pORF3 (31). Ribulose bis-phosphate carboxylase was used as a loading control. (Right) Quantification of results from two independent experiments. Error bars denote standard deviations. GFP protein levels seen in the presence of NMD are included for comparison (stripes). (E) Protocol for examining if virus levels were affected when NMD levels were enhanced or repressed by UPF1 or U1D, respectively. PEMV2 or PEMV2Δp26, along with p14 and either Mock (-), UPF1, or U1D, was subjected to coagroinfiltration into N. benthamiana. At 48 hpi, total RNA was extracted and gRNA levels were quantified using RT-qPCR. Coinfiltrated p14 was used as a reference gene. Error bars denote standards of results deviation from 2 biological replicates, each consisting of 3 to 4 pooled leaves (*, *P < *0.05; **, *P < *0.01 [one-way ANOVA with multiple-comparison test]).

Since the RNA stabilization activity of p26 was not due to RNA silencing suppression, we hypothesized that the RNA-binding ability of p26 might confer protection against NMD by occluding UPF1 binding. An agroinfiltration-based NMD assay previously developed by Kertesz et al. (11) was used to determine whether the presence of p26 alone can protect NMD-sensitive transcripts. The GFP reporters used in this assay have been previously described (11, 22) and differ with respect to the composition and length of sequence downstream of the GFP ORF (Fig. 2A) as follows: GFP-3′UTR_PEMV_ contains the PEMV2 3′ UTR; GFP-L (long sequence downstream of the GFP ORF) contains ∼500 nt from the bean phytohemagglutinin (PHA) gene; and GFP-S (short sequence downstream of the GFP ORF) contains only the CaMV 35S transcription termination sequence. Constructs with long 3′ UTRs (GFP-3′UTR_PEMV_ and GFP-L) are NMD sensitive, and GFP-S is NMD resistant (11, 22).

Three locations on individual N. benthamiana leaves were subjected to coagroinfiltration with p14, the GFP reporter construct, and mock treatment [Mock (-)] or treatment with a construct expressing p26 (+p26) or one expressing the NMD inhibitor U1D (+U1D) (Fig. 2B). U1D is a dominant-negative UPF1 bearing an arginine-to-cysteine mutation in the conserved RNA helicase domain (R863C) that strongly interferes with NMD (11). Background-adjusted GFP intensity was measured after 48 h in the infiltrated spots, and total RNA was isolated for Northern analysis and RT-qPCR. Compared to Mock samples, p26 increased accumulation of NMD-sensitive GFP-3′UTR_PEMV_ and GFP-L transcripts by 4-fold and 3-fold, respectively (Fig. 2B, bottom). Similarly, U1D increased accumulation of GFP-3′UTR_PEMV_ and GFP-L transcripts by 6.1-fold and 2.7-fold, respectively (Fig. 2B, bottom). In contrast, NMD-resistant GFP-S transcript levels were not enhanced by the presence of either p26 or U1D.

Interestingly, despite having similar mRNA levels, the intensity of GFP fluorescence produced by GFP-3′UTR_PEMV_ and GFP-L was lower in the presence of p26 than in the presence of U1D (Fig. 2B, top). Percentages of translational efficiencies (TE%) were determined by dividing the mean GFP intensities by the mean relative mRNA accumulation under each condition. In the presence of p26, GFP-3′UTR_PEMV_ and GFP-L had TE% values of 48% and 54%, respectively, while the presence of U1D had only a minor effect (Fig. 2B). Importantly, p26 did not decrease the TE% of NMD-resistant GFP-S transcripts, demonstrating that *in vivo*, p26 represses translation only of NMD targets with long 3′ UTRs and does not cause global translation inhibition (Fig. 2B). To determine if p26 directly inhibits translation in an *in vitro* assay, C-terminal histidine-tagged p26 (p26-His_6_) was preincubated (10:1 molar ratio) with transcripts from a previously described luciferase reporter construct (5′89 + 3U; contains the PEMV2 5′ 89 nt and 3′ UTR) (43), and translation was assayed for in wheat germ extracts (WGE). p26-His_6_ reduced translation of 5′89 + 3U by over 4-fold compared to buffer of otherwise identical composition (see Text S1 and Fig. S1A in the supplemental material). p26 also reduced translation of a polyadenylated control RNA that lacks a 3′ UTR (Fig. S1A), indicating that translation inhibition is not restricted to transcripts with PEMV2 sequences. Similar observations have been made for cellular RNA-binding proteins that render transcripts inaccessible to the translation machinery or interfere with translating ribosomes (44). Interestingly, p26 inhibited translation of both the NMD-resistant and NMD-sensitive reporters *in vitro*, but only the NMD-sensitive transcripts bearing long 3′ UTRs were translationally repressed *in vivo* (Fig. 2B).

10.1128/mBio.00204-20.2 FIG S1 p26 inhibits translation *in vitro* and *in vivo* and requires nucleolar localization for efficient systemic trafficking. (A) The 5′89 + 3U reporter contains the first 89 nt of PEMV2 gRNA fused in-frame with firefly luciferase preceding the PEMV2 3′ UTR. (Bottom left) Histidine-tagged p26 (p26-His_6_) was synthesized in WGE, purified, and detected by Western blotting using anti-His_6_ antibody. (Bottom right) p26-His_6_ was preincubated for 30 min with the reporter RNAs shown before translation in WGE. After 30 min of translation, luciferase expression was measured in a luminometer. Error bars denote standard deviations of results from at least two independent experiments and 6 replicates. (B) TMV-p26-GFP was capable of systemic trafficking, whereas TMV-p26-RN-GFP was severely hampered in systemic movement. Fluorescence of whole infected plants (top) and of systemic leaves of the same age (bottom) is indicated. (C) Translation of GFP-3′UTR_PEMV_ subjected to mock treatment (-) or treated with coinfiltrated HA-p26 (p26), HA-p26-RN (RN), or U1D. (Top) Relative GFP fluorescence intensity levels were measured using ImageJ (with mock treatment value set at 1.0). Error bars denote standard deviations of results from 7 separate leaves. Translational efficiencies (TE%) were calculated by dividing the mean GFP intensity by the mean mRNA accumulation under each condition, with mock treatment (-) value set at 100%. (Bottom) Western blotting was performed, and p26 was detected using anti-HA antibody (see legend for Fig. 2D). Data representing monomers (M) and dimers (D) are shown. Download FIG S1, TIF file, 1.0 MB. Copyright © 2020 May et al. 2020 May et al. This content is distributed under the terms of the Creative Commons Attribution 4.0 International license.

To determine if p26 can also protect transcripts from non-NMD degradation pathways (i.e., degradation pathways that are active in the presence of U1D), N. benthamiana leaves were subjected to infiltration with p14, GFP-3′UTR_PEMV_, U1D, and either Mock (-) or human influenza virus hemagglutinin (HA)-tagged p26 (HA-p26). In the presence of U1D, HA-p26 increased GFP-3′UTR_PEMV_ mRNA levels by 70% (versus 300% with active NMD), suggesting that p26-mediated protection is mainly but not exclusively associated with NMD (Fig. 2C). Alternatively, it remains possible that the 70% increase in GFP-3′UTR_PEMV_ mRNA levels seen with HA-p26 expression is attributable to incomplete NMD inhibition by U1D. While HA-p26 increased mRNA levels by 70%, translation of GFP was not significantly increased (Fig. 2D), supporting the proposal that p26 was reducing the translational efficiency of NMD-sensitive transcripts.

If p26 is primarily responsible for protecting viral transcripts from NMD, then a disproportional increase in virus accumulation should occur when PEMV2Δp26 is coinfiltrated with U1D. However, the relative (2-fold) increase in PEMV2Δp26 gRNA levels seen under conditions of NMD inhibition (+U1D) was comparable to the level seen with the WT (Fig. 2E) and was not disproportionately higher (i.e., 4-fold) than that required to reach absolute levels equal to the WT levels (Fig. 2B). This suggests that NMD inhibition alone is insufficient to compensate for the deleterious effects of the loss of p26 expression, demonstrating that p26 is a multifunctional protein involved in additional aspects of PEMV2 replication aside from NMD protection and long-distance trafficking.

### p26 confers NMD resistance exclusively from the cytoplasm.

**FIG 3:**
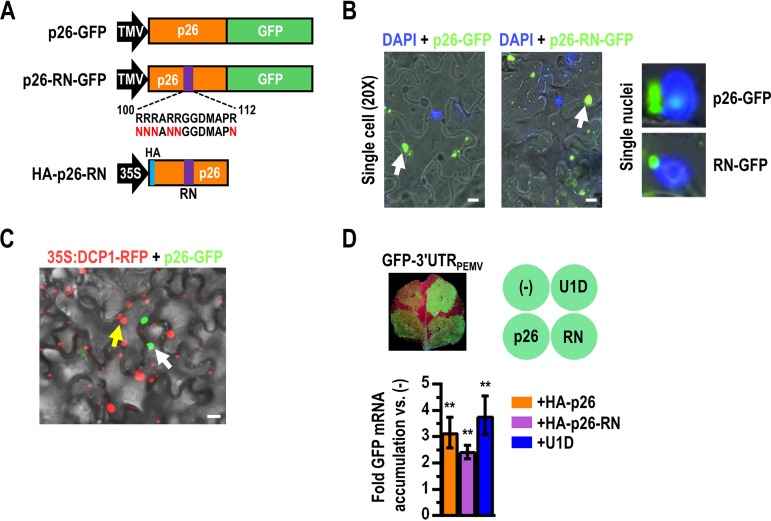
p26 protects NMD-sensitive transcripts exclusively in the cytoplasm. (A) TMV expression constructs for p26 fused to GFP (p26-GFP). Six arginines within p26 were altered to asparagines, generating p26-RN, to eliminate nucleolar localization. Infiltration-competent TMV vectors designed to express either p26-GFP or p26-RN-GFP were used for p26 visualization. (B) (Left two panels) Fluorescence microscopy of leaves infiltrated with p14 and TMV expressing either p26-GFP or p26-RN-GFP. Arrows point to large cytoplasmic inclusion bodies formed by both p26-GFP and p26-RN-GFP (bars, 10 μm). (Right) Digital zoom from ×20 magnification images showing single nuclei. In ∼1% of cells, p26-GFP was also observed in the nucleolus (top), whereas p26-RN-GFP was exclusively cytoplasmic (bottom). (C) N. benthamiana cells systemically infected with TMV expressing p26-GFP were subjected to agroinfiltration with P-body marker DCP1-RFP in upper leaves to visualize P-bodies and p26 inclusion bodies in the same cells. The white arrow denotes p26 inclusion bodies, and the yellow arrow denotes a P-body. Bar, 10 μm. (D) HA-p26 and HA-p26-RN were assayed for the ability to protect GFP-3′UTR_PEMV_ from NMD. (Top) Leaves were subjected to infiltration with p14, GFP-3′UTR_PEMV_, and HA-p26, HA-p26-RN, or U1D. (Bottom) After 48 hpi, total RNA was extracted to assay for GFP mRNA levels by RT-qPCR. Error bars denote standard errors. One-way ANOVA was used with a multiple-comparison test to determine the statistical significance of data representing increase over leaves infiltrated with only p14 and GFP-3′UTR_PEMV_ (5 independent leaves) (**, *P < *0.01).

Long-distance movement of GRV gRNA requires intracellular transport of pORF3 to the nucleolus for interaction with fibrillarin nucleolar protein (32, 33). To determine if nucleolar localization of p26 is required for protection of NMD-sensitive transcripts, 6 arginines that are necessary for nucleolar localization of pORF3 (45) and conserved in p26 (Fig. S2) were mutated to asparagine, generating “RN”-designated mutants (Fig. 3A). WT p26 and p26-RN were C-terminally fused with GFP and inserted in place of the capsid protein ORF in a TMV expression vector that generates TMV gRNA that is incapable of systemic movement (46). TMV expressing either p26-GFP or p26-RN-GFP was subjected to infiltration into N. benthamiana leaves, and local leaves were imaged by fluorescence microscopy 5 to 7 days later. Both p26-GFP and p26-RN-GFP formed large cytoplasmic inclusion bodies in infected cells that were similar in size to those seen in DAPI (4′,6-diamidino-2-phenylindole)-stained nuclei (Fig. 3B, left two panels). The vast majority of p26-GFP and p26-RN-GFP was observed in the cytoplasm, supporting the earlier report that in ∼97% of cells, GRV pORF3-GFP was detected only in the cytoplasm (45). However, in ∼1% of cells, p26-GFP localized to the nucleus, whereas p26-RN-GFP was always exclusively cytoplasmic/perinuclear (Fig. 3B, right). Coexpression of p26-GFP and red fluorescent protein (RFP)-tagged DCP1 (a P-body marker) revealed that the p26 inclusion bodies were spatially separate from the P-bodies (Fig. 3C), which are considered to be major sites of NMD decay in plants (6, 7). Only p26-GFP was able to efficiently complement the defect in systemic movement of the TMV vector (Fig. S1B), confirming the previously reported detrimental effect of the pORF3 RN mutations on systemic movement of GRV (45).

10.1128/mBio.00204-20.3 FIG S2 Conservation of the arginine-rich motif between PEMV2 p26 and GRV pORF3. Umbravirus pORF3 amino acid sequences were first aligned using a progressive alignment method and pairwise distances were calculated to construct a phylogenetic tree. Six conserved arginine residues were mutated to asparagine (red arrows) in PEMV p26 to generate p26-RN. Accession numbers for umbravirus pORF3 amino acid sequences are as follows: YP_009162615.1 (OPMV, *Opium poppy mosaic virus*), NP_733849.1 (TBTV, *Tobacco bushy top virus*), YP_009056850.1 (ETBTV, *Ethiopian tobacco bushy top virus*), NP_619660.1 (GRV, *Groundnut rosette virus*), NP_620847.1 (PEMV2, *Pea enation mosaic virus 2*), NP_054008.1 (CMoMV, *Carrot mottle mimic virus*), and AHA85539.1 (CMoV, *Carrot mottle virus*). Download FIG S2, TIF file, 0.5 MB. Copyright © 2020 May et al. 2020 May et al. This content is distributed under the terms of the Creative Commons Attribution 4.0 International license.

To determine if HA-p26-RN was still able to protect transcripts from NMD despite an apparent inability to enter the nucleus, leaves were subjected to infiltration with p14 or GFP-3′UTR_PEMV_ and with HA-p26, HA-p26-RN, or U1D (Fig. 3D). GFP-3′UTR_PEMV_ transcript levels were 3.1-fold and 2.4-fold higher under conditions of coexpression with HA-p26 and HA-p26-RN, respectively, than those seen under conditions of mock treatment, suggesting that nucleolar localization of p26 and long-distance movement of p26-bound RNAs are not required for NMD resistance. Addition of U1D to inhibit NMD resulted in a comparable (3.7-fold) increase in GFP-3′UTR_PEMV_ transcript levels (Fig. 3D). As before, the TE% of the GFP-3′UTR_PEMV_ reporter was reduced in the presence of either HA-p26 or HA-p26-RN (Fig. S1C).

### Transcriptome-wide analyses revealed that p26 protects a subset of host NMD targets and that NMD is impaired during PEMV2 infection.

Since p26 protected both viral and nonviral mRNAs from NMD, we examined whether p26 generated transcriptome-wide alterations by performing transcriptome sequencing (RNA-seq) using infiltration with the following combinations: (i) p14 and an equivalent amount of C58C1 agrobacteria (Mock); (ii) p14 and U1D; (iii) p14 and HA-p26; and (iv) p14 and PEMV2. A high degree of clustering was observed by principal-component analysis under each set of conditions (Fig. S3A). Differential gene expression analysis identified thousands of genes that had been both downregulated (Down) and upregulated (UP) under each set of conditions, with PEMV2 causing the most substantial changes in gene expression (Fig. S3B; see also Data Set S1 in the supplemental material).

10.1128/mBio.00204-20.4 FIG S3 RNA-seq experimental design. (A) N. benthamiana leaves were subjected to infiltration with p14 and empty C58C1 agrobacteria (Mock), U1D, HA-p26, or PEMV2 virus. Total RNA was used to prepare an RNA-seq library. Principal-component analysis (PCA) showed a high degree of clustering for grouped samples. (B) Differential gene expression was present under all conditions tested. A negative binomial model was used to calculate adjusted *P* values (false-discovery rate [FDR]). (C) Total RNAs from Mock and +PEMV2 samples separated by agarose gel electrophoresis. Note that PEMV2 gRNA accumulates to rRNA levels when RNA silencing is suppressed. Download FIG S3, TIF file, 1.7 MB. Copyright © 2020 May et al. 2020 May et al. This content is distributed under the terms of the Creative Commons Attribution 4.0 International license.

10.1128/mBio.00204-20.8 DATA SET S1 Processed RNA-seq data determined under all conditions. The Data Set includes 3’ UTR sequence, GC% content, and uORF data for each transcript. Download Data Set S1, XLSX file, 19.1 MB. Copyright © 2020 May et al. 2020 May et al. This content is distributed under the terms of the Creative Commons Attribution 4.0 International license.

**FIG 4:**
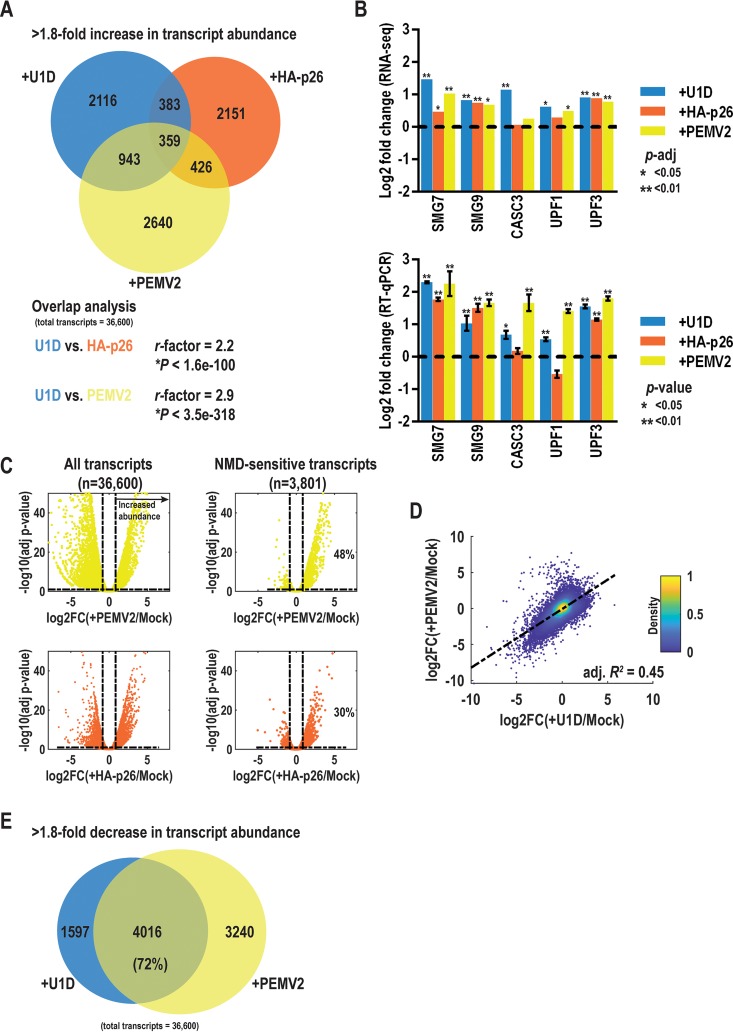
Significant overlap of NMD inhibition, p26 expression, and PEMV2 infection in host transcripts with increased abundance. (A) RNA-seq was used to identify host transcripts with a >1.8-fold increase in transcript abundance when NMD was inhibited with U1D (+U1D) or when HA-p26 was expressed or during PEMV2 infection. The representation factor (*r-*factor) represents the number of shared genes divided by the expected number of shared genes from two independent groups, where a value of >1 indicates a higher degree of overlap than expected. (B) Identities of reviously validated NMD targets were confirmed with RNA-seq (top) and RT-qPCR (bottom) under the three experimental conditions. RT-qPCR data represent results from three independent biological replicates, and gene expression was normalized to either actin (+U1D and +PEMV2) or p14 (+HA-p26). *P* values were calculated using an unpaired *t* test for comparisons between the mock treatment group (Mock) and the experimental group. *p*-adj, adjusted *P* value. (C) Volcano plots for all transcripts (left) or NMD-sensitive transcripts (right) during PEMV2 infection (yellow) or HA-p26 expression (orange). Vertical dashed lines represent a 1.8-fold increase or decrease in transcript abundance. The horizontal dashed line denotes a 10% false-discovery rate (FDR; adjusted *P* value = 0.1). Percentage values denote the portion of transcripts with significantly increased abundance and <10% FDR. (D) Log_2_ fold change (log2FC) values compared to mock treatment (Mock) were plotted for +PEMV2 and +U1D. Bar colors to the right represent the density of transcripts. Unbiased, adjusted *R*-squared values represent the goodness of fit for a linear regression model. +PEMV2 and +U1D data displayed a positive linear correlation, with 45% of the variance explained by the tested model. (E) Data representing off-target transcripts with a >1.8-fold decrease in abundance under conditions of NMD inhibition (+U1D) or of PEMV2 infection have a high degree of overlap. A total of 72% of downregulated transcripts with U1D expression were also downregulated during PEMV2 infection.

RNA-seq has been used to reliably detect 1.8-fold changes in gene expression by using at least 3 biological replicates and modeling count data as a negative binomial distribution (47). Transcripts whose levels increased >1.8-fold when NMD was inhibited by U1D were considered possible NMD targets (Fig. 4A, blue) (*n* = 3,801). Approximately 10% of the unique transcripts detected in this study showed >1.8-fold increases in transcript abundance with U1D, which is similar to what was observed with NMD inhibition mediated by upf1 knockdown or knockout in multiple eukaryotic systems (48–52). Twenty percent of transcripts whose abundance increased >1.8-fold with NMD inhibition also showed >1.8-fold-increased abundance with HA-p26, and 34% of these NMD-sensitive transcripts showed >1.8-fold-increased abundance during PEMV2 infection (Fig. 4A). *Arabidopsis* NMD targets were previously found to be involved in diverse biological processes (50). Similarly, GO term enrichment analysis of transcripts upregulated >1.8-fold under all three sets of conditions resulted in few enriched processes but included macromolecule and protein modification as well as lipid and protein metabolism (Text S1 and Fig. S4; see also Data Set S2). Components of the NMD pathway whose transcripts are degraded by NMD as part of an autoregulatory system (SMG7, SMG9, UPF1, and UPF3) and EJC component CASC3 (53, 54) were selected for further RT-qPCR analysis. NMD inhibition (+U1D) and PEMV2 infection resulted in increased transcript levels for SMG7, SMG9, UPF1, UPF3, and CASC3, whereas HA-p26 expression increased transcript levels only for SMG7, SMG9, and UPF3 (Fig. 4B). This suggests that HA-p26 and, to a greater extent, PEMV2 virus protect natural NMD targets from decay.

10.1128/mBio.00204-20.5 FIG S4 Gene Ontology (GO) term enrichment analysis for transcripts upregulated with NMD inhibition, HA-p26 expression, and PEMV2 infection. N. benthamiana GO annotations were used for singular enrichment analysis (SEA) using AgriGO v2 (2). The biological processes that were significantly enriched (*P < *0.01) for upregulated transcripts are shown with their corresponding *P* values and GO term identifiers (IDs). Download FIG S4, TIF file, 1.1 MB. Copyright © 2020 May et al. 2020 May et al. This content is distributed under the terms of the Creative Commons Attribution 4.0 International license.

10.1128/mBio.00204-20.9 DATA SET S2 GO term analysis for transcripts upregulated with HA-p26, U1D, and PEMV2 expression. Download Data Set S2, XLSX file, 0.04 MB. Copyright © 2020 May et al. 2020 May et al. This content is distributed under the terms of the Creative Commons Attribution 4.0 International license.

Although PEMV2 infection and HA-p26 expression resulted in upregulation and downregulation of thousands of transcripts (Fig. 4C, left), the NMD-sensitive transcripts (i.e., the transcripts significantly upregulated in the presence of U1D) were heavily skewed toward increased abundance for both (Fig. 4C, right). Using a 10% false-discovery rate, 1,820 (48%) of the natural NMD targets showed significantly increased abundance during PEMV2 infection (Fig. 4C, yellow), and 1,140 (30%) showed significantly increased abundance with HA-p26 expression (Fig. 4C, orange). These results demonstrate that a significant portion of natural NMD targets are protected during PEMV2 infection and that many are protected by p26 alone.

Since inhibition of NMD by UPF1 knockdown or U1D overexpression results in specific off-target effects (i.e., transcripts whose expression decreased >1.8-fold with U1D expression) (53, 55), we hypothesized that PEMV2-mediated NMD inhibition would display similar off-target effects. Plotting the log_2_ fold change (log2FC) values representing results of comparisons of transcript abundances between +PEMV2 and +U1D samples revealed a linear correlation (Fig. 4D). Among the transcripts whose expression decreased >1.8-fold with U1D expression, 72% showed significantly decreased expression during PEMV2 infection (Fig. 4E). In contrast, no linear correlation was observed in comparisons of expression profiles between +HA-p26 and either +U1D or +PEMV2 (Fig. S5A and B). The lack of any correlation between the expression profiles of +HA-p26 and +U1D suggests that the presence of p26 alone does not cause global NMD inhibition but instead protects a defined subset of NMD targets. Together, these findings demonstrate that PEMV2 infection disrupts the NMD pathway and causes off-target effects similar to those caused by a known NMD inhibitor.

10.1128/mBio.00204-20.6 FIG S5 Host transcript expression profiles corresponding to +HA-p26/+U1D and +HA-p26/+PEMV2 display a low degree of correlation. Log2 fold changes (log2FC) compared to mock treatment were plotted for +HA-p26/+U1D (A) and +HA-p26/+PEMV2 (B). The bar colors to the right represent the density of transcripts. Unbiased, adjusted *R*-squared values represent the goodness of fit for a linear regression model. Download FIG S5, TIF file, 1.9 MB. Copyright © 2020 May et al. 2020 May et al. This content is distributed under the terms of the Creative Commons Attribution 4.0 International license.

### HA-p26 preferentially upregulates transcripts with long, GC-rich, structured 3′ UTRs.

**FIG 5:**
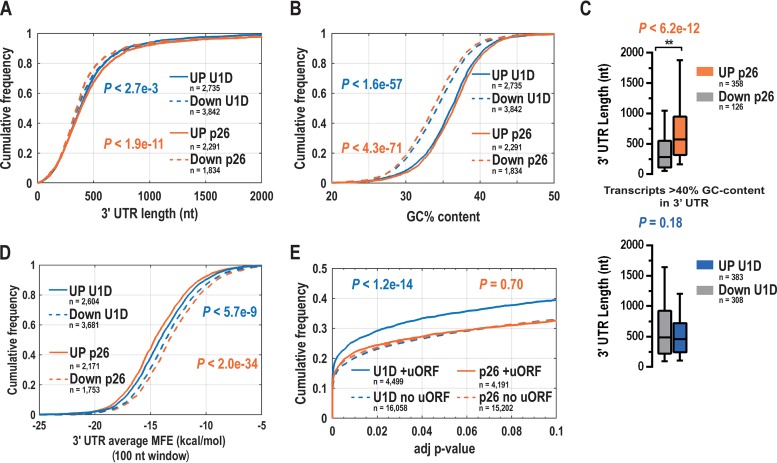
HA-p26 protects transcripts with GC-rich 3′ UTRs. (A) 3′ UTR lengths of transcripts that were either upregulated (NMD target) or downregulated (non-NMD target) with U1D or HA-26 were examined using a cumulative distribution function (CDF) plot. *P* values represent the significance level of data from a two-sample Kolmogorov-Smirnov test for each data set. (B) CDF plots representing the GC% content of 3′ UTRs from upregulated and downregulated transcripts with U1D or p26 expression. (C) Box plots represent the 3′ UTR lengths for transcripts with >40% 3′ UTR GC content. The horizontal line represents the median, while the upper and lower box edges represent the 90th and 10th percentiles, respectively. A value of >1.8-fold change in abundance was used as a cutoff for both upregulated and downregulated transcripts. *P* values are from a two-sided Kruskal-Wallis test performed to determine whether samples originated from the same distribution. (D) Minimum folding energies (MFE) of the 3′ UTRs (100-nt window size) of upregulated and downregulated transcripts were calculated using RNAslider (77). (E) Upstream ORFs (uORFs) were identified in annotated N. benthamiana transcripts using getorf (emboss). CDF plots for adjusted *P* values for transcripts with >0.9-fold change are shown for transcripts with and without uORFs from the U1D and HA-p26 RNA-seq data sets.

Since the 3′ UTR is a major determinant of NMD sensitivity, we analyzed the 3′ UTRs of transcripts that were found to have increased in abundance upon NMD inhibition or HA-p26 expression (see Data Set S1). Despite the availability of only limited data from reporter assays showing a link between 3′ UTR length and NMD sensitivity (Fig. 2B), examination of the entire transcriptome showed that the transcripts that were increasing in abundance in the presence of U1D did not have significantly longer 3′ UTRs (Fig. 5A). Similar observations were made in previous transcriptome-wide analyses of NMD targets (5). Transcripts with increased abundance with HA-p26 expression had slightly longer 3′ UTRs than downregulated transcripts (Fig. 5A). Both NMD targets (UP U1D) and transcripts protected by HA-p26 (UP p26) had 3′ UTRs that showed significantly higher GC-rich levels than were seen with the downregulated genes (Down U1D and Down p26) (Fig. 5B). Transcripts with both GC-rich (>40%) and long 3′ UTRs were preferentially upregulated by HA-p26, suggesting that the 3′ UTR length is a determining factor for p26-mediated NMD resistance (Fig. 5C, top). Interestingly, this effect was not observed with U1D (Fig. 5C, bottom). In addition, transcripts protected by HA-p26 had significantly greater levels of secondary structure than were seen with U1D (Fig. 5D).

Upstream ORFs (uORFs) promote NMD, since translation termination of the uORF results in an extended 3′ UTR (56). Predicted uORFs were identified for annotated N. benthamiana transcripts by the use of a minimum ORF length of 30 amino acids (Data Set S1). Examining transcripts with a >0.9-fold change in the presence of U1D, uORF-containing transcripts had significantly lower adjusted *P* values, demonstrating that uORFs represent a potent NMD-inducing feature (Fig. 5E). In contrast, HA-p26 expression did not result in increased transcript abundance for uORF-containing transcripts (Fig. 5E). These results suggest that p26 and U1D have different modes of conferring NMD resistance.

Differential motif enrichment analysis using MEME (57) was also performed for comparisons between NMD-resistant transcripts (i.e., Down U1D) and UP U1D or UP p26 transcripts. Only the first 100 nt of the 3′ UTRs were examined since the region immediately downstream of the stop codon is the primary location for NMD-resistant motifs (22, 58). The only motif whose level was significantly enriched was a polypyrimidine hexamer among NMD-resistant transcripts (Fig. S6). This observation supports earlier work demonstrating that polypyrimidine tracts near a PTC can bind PTBP1 and confer NMD resistance (21). Altogether, these results suggest that HA-p26 preferentially upregulates transcripts with long, GC-rich, structured 3′ UTRs but does not differentially protect transcripts with uORFs.

10.1128/mBio.00204-20.7 FIG S6 Polypyrimidine hexamers are enriched downstream of stop codons in NMD-resistant transcripts. The first 100-nt sequence of the 3′ UTRs of transcripts with a >1.8-fold increase or decrease in the presence of U1D or a >1.8-fold increase with HA-p26 was extracted for differential motif enrichment analysis using MEME (3). A polypyrimidine hexamer was the only enriched sequence in NMD-resistant transcripts (Down U1D) compared to transcripts with increased abundance with either U1D or HA-p26. Download FIG S6, TIF file, 0.3 MB. Copyright © 2020 May et al. 2020 May et al. This content is distributed under the terms of the Creative Commons Attribution 4.0 International license.

## DISCUSSION

RNA viruses must protect their viral genomes from RNA degradation pathways for successful amplification. NMD is triggered by long 3′ UTRs (>300-nt-long 3′ UTRs in N. benthamiana [11]), making most plant RNA viruses susceptible to this degradation pathway. Despite a fundamental requirement to circumvent NMD, little is known about how plant RNA viruses protect their genomes. TCV and other carmoviruses contain a ribosome readthrough recoding element that likely protects their gRNA and a short (∼50-nt) unstructured region that protects their coat protein/silencing suppressor-encoding 1.45-kb sgRNA (22). The PEMV2 frameshift recoding site also likely confers some protection to the gRNA, and p26 binding to the region downstream of p94 may confer additional protection. During natural infections, enamovirus PEMV1 (GenBank accession no. NC_003629) RNA would be accessible to p26 and, as seen with PEMV2, translation termination after the PEMV1 RNA-dependent RNA polymerase ORF leaves a 1,896-nt 3′ UTR. Therefore, p26 might be beneficial for PEMV1 during coinfections by protecting PEMV1 gRNAs from NMD. Unlike results observed previously for some carmoviruses (22), no *cis*-acting sequences protect the critical PEMV2 sgRNA, which encodes p26 and p27. We suggest that a novel form of protection of viral and host mRNAs against NMD is conferred by the *trans*-acting p26 long-distance MP on the basis of the following findings. (i) p26 expression alone protected NMD-sensitive transcripts from NMD (Fig. 2B). (ii) A significant number of NMD-susceptible host transcripts with long, GC-rich 3′ UTRs increased in accumulation in the presence of p26 or PEMV2 (Fig. 4). *In vivo*, p26 repressed translation only of NMD-sensitive transcripts with long 3′ UTRs (Fig. 2B) but repressed translation of all tested transcripts *in vitro* (see Fig. S1A in the supplemental material). A possible explanation is that under conditions of p26 preincubation with transcripts in the absence of any translation machinery *in vitro*, the entire transcript is accessible for binding. However, the NMD-resistant transcripts are actively translating *in vivo* and are mostly bound as polysomes, and the short 3′ UTR may be insufficient for p26 association. A similar observation was previously reported regarding UPF1 binding (12), where long 3′ UTRs were found to be heavily associated with UPF1 *in vivo* whereas short 3′ UTRs were shown to be insufficient for UPF1 binding. Translational repression of transcripts with the PEMV2 3′ UTR could also result from disruption of 3′ cap-independent translation elements (CITEs) that are required for translation initiation by all *Tombusviridae* (59). Additional studies will be required to determine if the RNA secondary structures and/or functions of 3′ CITEs are perturbed in the presence of p26.

**FIG 6:**
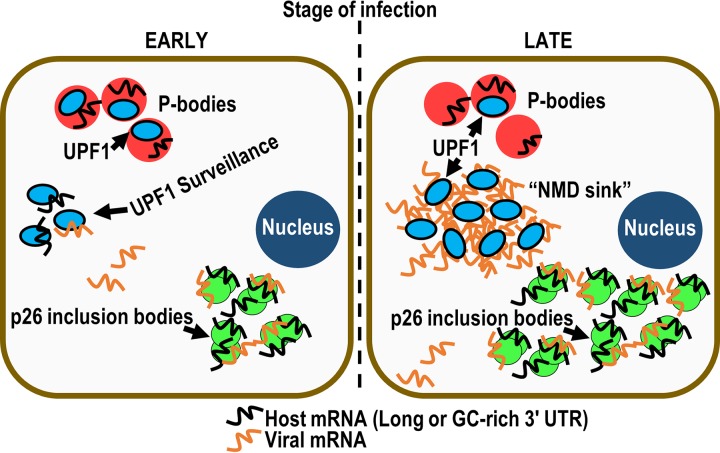
Model for p26-mediated and PEMV2-mediated NMD resistance. During the early stages of infection (left), p26 inclusion bodies bind viral and host mRNAs and shield them from UPF1 surveillance. Late in the infection (right), the high levels of viral RNAs bind and sequester UPF1 (and possibly additional NMD factors), creating an “NMD sink.” As a result, UPF1 surveillance is severely impaired, resulting in increased abundance of transcripts normally targeted by NMD.

On the basis of previous results showing that the GRV pORF3 orthologue efficiently binds single-stranded RNA (ssRNA), double-stranded RNA (dsRNA), single-stranded DNA (ssDNA), and tRNA with no apparent specificity for its cognate transcripts (31) and showing the ability of pORF3 and p26 to form large inclusion bodies (31) (Fig. 3B and C), we propose that both viral and host mRNAs are bound by p26 in cytoplasmic inclusion bodies and that these transcripts are protected from NMD surveillance (Fig. 6, left). It remains unclear if p26 association with viral RNAs is reversible and whether viral RNAs in p26 inclusion bodies are replication competent (i.e., accessible to the RNA-dependent RNA polymerase and other components necessary for replication). MPs from diverse plant viruses may also confer NMD resistance to viral and host transcripts in addition to virus trafficking. Plant virus MPs in general are nonspecific RNA-binding proteins and thus may also provide protection to noncognate RNAs. In addition, the dianthovirus *Red clover necrotic mosaic virus* contains a 35-kDa MP that forms RNase-resistant RNP complexes (60), which would likely also be immune to UPF1 surveillance.

Using a transcriptome-wide approach, we demonstrated that both PEMV2 infection and expression of p26 alone protect a substantial (up to 48%) portion of NMD-targeted transcripts. Due to the high degree of overlap of upregulated and downregulated transcripts during PEMV2 infection and U1D-mediated NMD inhibition (Fig. 4), we suggest that NMD inhibition is a major factor in driving the transcriptome-wide changes in gene expression observed during PEMV2 infection. Interestingly, genes involved in lipid metabolism were disproportionately upregulated upon NMD inhibition, p26 expression, or PEMV2 infection (Fig. S4). Tombusviruses have been shown to modulate lipid metabolism to promote the assembly of virus replicase complexes on peroxisome membranes (61–63). In the present study, transcripts encoding the class III peroxidase superfamily proteins *PER4* and *PER15* (64), as well as the critical peroxisome biogenesis protein *PEX12* (65), were upregulated under all conditions (see Data Set S2 in the supplemental material). These data suggest that NMD inhibition by PEMV2 could benefit virus replication by increasing peroxisome membrane biogenesis to support increasing amounts of virus replicase formation.

Earlier studies demonstrated that UPF1 targets transcripts with GC-rich 3′ UTRs (9, 10), and both U1D and p26 were identified as preferentially protected transcripts with GC-rich 3′ UTRs in the present study (Fig. 5B). p26 expression alone led to increased transcript abundance for 30% of host NMD targets (versus 48% during PEMV2 infection), suggesting that PEMV2 may have additional anti-NMD properties. In silencing-suppressed N. benthamiana, PEMV2 gRNA accumulates to rRNA levels (Fig. S3C) and the extraordinary amount of viral RNA in the late stages of an infection might also enhance NMD inhibition by serving as a sink for binding to and sequestering UPF1 and possibly additional NMD factors (Fig. 6, right). Considering the amount of PEMV2 RNA present during an established infection, endogenous UPF1 is unlikely to have any substantial effect on PEMV2 accumulation; however, NMD surveillance would be severely impaired due to a lack of free UPF1 (Fig. 6, right).

Viral proteins from HTLV-1, MHV, ZIKV, HCV, and CaMV confer NMD resistance by directly targeting NMD or RNA decay factors (e.g., UPF1 or the EJC) (15, 16, 25, 29, 30). However, evidence of viral proteins directly binding to viral (or nonviral) transcripts and conferring NMD resistance is limited. In contrast, several host proteins confer NMD resistance when bound immediately downstream of a NMD-sensitive stop codon. PABPC1 binds poly(A) tracts and inhibits NMD when tethered immediately downstream of a PTC (66), while PTBP1 blocks UPF1 association by binding polypyrimidine tracts in the same region (21). Similarly, hnRNP L confers NMD resistance by binding CA repeats downstream of NMD-targeted stop codons (67). p26 may function in a similar manner to protect some NMD targets by binding downstream of an NMD-targeted stop codon and preventing UPF1 association.

In summary, this study demonstrated that the PEMV2 long-distance-movement protein is multifunctional and protects both cellular and viral transcripts from NMD. Furthermore, the NMD pathway as a whole is largely dysfunctional during PEMV2 infection and that disfunctionality could be the driving force behind the large-scale changes in gene expression observed during virus infections. It remains unclear if RNA viruses directly benefit from host gene expression changes resulting from NMD inhibition, and future work will be aimed at examining the interplay between RNA virus replication and NMD inhibition.

## MATERIALS AND METHODS

### Expression constructs.

pCB-PEMV2 and pCB-PEMV2Δp26 full-length expression plasmids were constructed by inserting cDNA into full-length PEMV2 gRNA downstream of the CaMV 35S promoter in the binary plasmid pCB301 by ligation-independent cloning (22, 68). The hepatitis delta virus (HDV) ribozyme was positioned at the 3′ terminus to generate authentic 3′ ends. The PZP-GFP and PZP-p19 expression constructs used for RNA silencing suppressor detection have been previously described (69). PEMV2 p26 and p33 ORFs were cloned into pRTL2 using the NcoI and XbaI restriction sites, and the entire expression cassette transferred to binary vector pPZP212 after excision and ligation using PstI. Additional constructs used in the transient NMD assay (UPF1, U1D, GFP-L, GFP-S, and GFP-3UTR_PEMV_) have been previously described (11, 22). Untagged and N-terminally HA-tagged p26 expression constructs were generated by cloning the respective PCR fragments into the BamHI and SalI restriction sites in pBIN61 (70). For studying p26 localization, p26-GFP fusion fragments with 5′ and 3′ PacI and NotI restriction sites, respectively, were generated by overlap-extension PCR and subsequently cloned into pJL-TRBO (46) using the corresponding restriction sites.

### Agroinfiltration.

Agrobacterium tumefaciens strain C58C1 or GV3101 (for PZP constructs) was transformed with expression constructs and cultured in the presence of antibiotics. After 2 days (passaged after day 1), pellets were resuspended in resuspension buffer (10 mM MgCl_2_, 10 mM morpholineethanesulfonic acid [MES]-K [pH 5.6], 100 μM acetosyringone). For all infiltrations, a final optical density at 600 nm (OD_600_) of 0.2 was used for p14, UPF1, U1D, GFP reporters, viral proteins (e.g., p26 and p33), and full-length viruses, unless otherwise noted. Mixtures were incubated at room temperature for at least 2 h prior to infiltration of N. benthamiana (fourth to fifth leaf stage). GFP fluorescence was observed using long-wave UV light after 48 h, and background-adjusted GFP intensity was measured using ImageJ.

### RNA analysis.

TRIzol (Invitrogen) was used to isolate total RNA from infiltrated “spots” in N. benthamiana leaves. For Northern blotting, RNAs were separated by agarose gel electrophoresis and transferred to a charged nylon membrane by capillary action in 10× MOPS [3-(*N*-morpholino)propanesulfonic acid]. After UV cross-linking, membranes were hybridized with [α-^32^P]dATP-labeled DNA probes (cocktail consisting of three 15-nt to 20-nt oligonucleotides). Reverse transcription-quantitative PCR (RT-qPCR) was first performed by first treating total RNA samples with RQ1 DNase (Promega). Next, 80-bp to 200-bp PCR fragments were amplified using a Luna one-step RT-qPCR kit (New England BioLabs). Reference genes included the p14 silencing suppressor (if infiltrated) or actin (ACT2). Relative fold changes were calculated using the threshold cycle (2^−ΔΔ^*^CT^*) method. All primers were designed using Primer3 online software (71).

### Western blotting.

N. benthamiana leaves expressing proteins of interest were ground in liquid nitrogen and resuspended in 10× phosphate-buffered saline (PBS)–2% β-mercaptoethanol. Lysates were resolved by 10% SDS-PAGE and transferred by a semidry transfer method. For loading controls, ribulose bis-phosphate carboxylase was imaged using stain-free gels (Bio-Rad). Primary antibodies targeting GFP (BioVision, Inc.), HA epitope (Rockland Immunochemicals Inc.), or His_6_ tag (GenScript) were used at a 1:10,000 dilution. Secondary antibody (horseradish peroxidase [HRP]-conjugated goat anti-rabbit IgG) was used at a dilution of 1:10,000. Chemiluminescence was detected using SuperSignal West Pico substrate (Thermo Scientific).

### Fluorescence microscopy.

N. benthamiana leaves were subjected to infiltration with p26-GFP fusion constructs in the pJL-TRBO vector (46) at an OD_600_ of 0.4. After strong GFP fluorescence was observed with long-wave UV light (typically 5 to 7 days), thin epidermal layers of leaves were removed and stained with 2 μg/ml of 4′,6-diamidino-2-phenylindole (DAPI). Leaf samples were imaged using a Nikon Eclipse microscope with Nikon DS-Qi1Mc light detector and a 20× lens objective. To observe p26 and DCP1 (P-body marker) localization, 35S:DCP1-RFP (kind gift of Alexis Maizel) was subjected to agroinfiltration into upper leaves of plants systemically infected with TRBO-p26-GFP. At 3 dpi, whole-leaf sections were mounted for microscopy. DCP1 and p26 localization was observed using a laser scanning LSM 710 AxioObserver confocal microscope (Carl Zeiss). GFP and RFP filters were used with a 10× lens objective.

### RNA-seq.

Agroinfiltration was performed using OD_600_ levels of 0.2 for p14 and 0.4 for C58C1 agrobacteria (Mock), U1D, HA-p26, or PEMV2 virus. After agroinfiltration (3 dpi), total RNA was isolated from the pooled fourth and fifth N. benthamiana leaves using a total RNA plant minikit (IBI Scientific), which included an in-column DNase I digestion step. Library preparation was performed by poly(A) selection followed by sequencing using an Illumina HiSeq system with a 2-by-150-bp configuration (Genewiz). The pipeline for RNA-seq analysis was as follows. (i) Trim Galore (http://www.bioinformatics.babraham.ac.uk/projects/trim_galore/) was used to remove Illumina adapter sequences and low-quality (Phred score of <20) reads. To avoid the presence of sequencing artifacts at the 5′ ends, the first 9 nucleotides were also removed. Sequence reads shorter than 20 nt or orphan reads were eliminated prior to a final quality check with FastQC. (ii) Cleaned reads were assigned to previously annotated N. benthamiana transcripts (ftp://ftp.solgenomics.net/genomes/Nicotiana_benthamiana/annotation/Niben101/) and quantified using Salmon (72). (iii) Differentially expressed genes were identified using a negative binomial model (73). A series of built-in and custom MATLAB scripts were used for bioinformatics analyses.

### Data availability.

Materials generated in this study (see Text S1 in the supplemental material) will be made available upon request. Both the raw sequencing data and processed read counts have been deposited in the Gene Expression Omnibus (GEO) database, under accession no. GSE137256.

10.1128/mBio.00204-20.1 TEXT S1 Supplemental materials and methods with corresponding references. Download Text S1, DOCX file, 0.01 MB. Copyright © 2020 May et al. 2020 May et al. This content is distributed under the terms of the Creative Commons Attribution 4.0 International license.
