# Supplementary figures and images for: The Multifunctional Long-Distance Movement Protein of Pea Enation Mosaic Virus 2 Protects Viral and Host Transcripts from Nonsense-Mediated Decay
Source: mBio. 2020 Mar 10;11(2):e00204-20. doi: 10.1128/mBio.00204-20 (PMC7064760; doi:10.1128/mBio.00204-20)

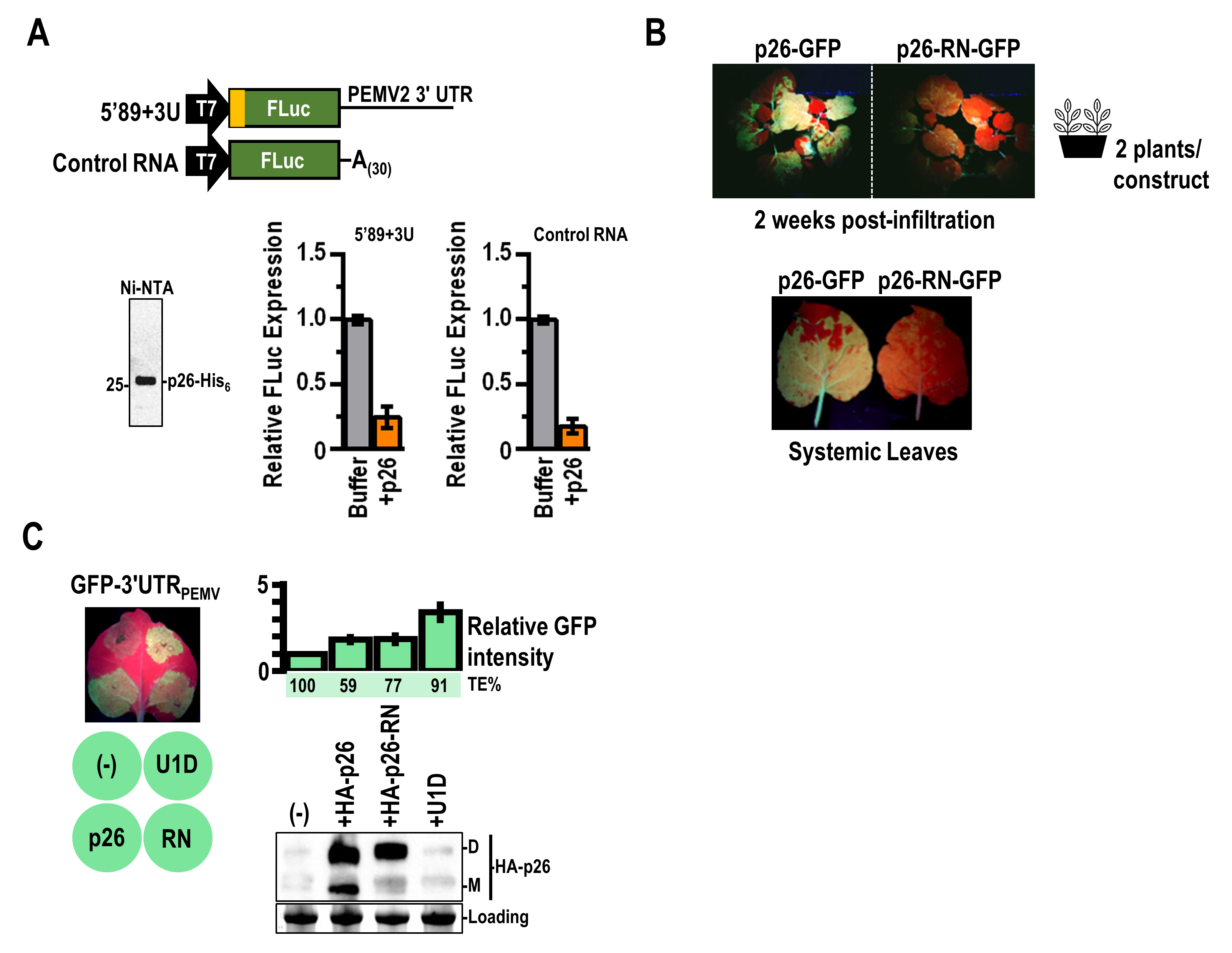

Supplement: FIG S1 [file mBio.00204-20-sf001.tif]

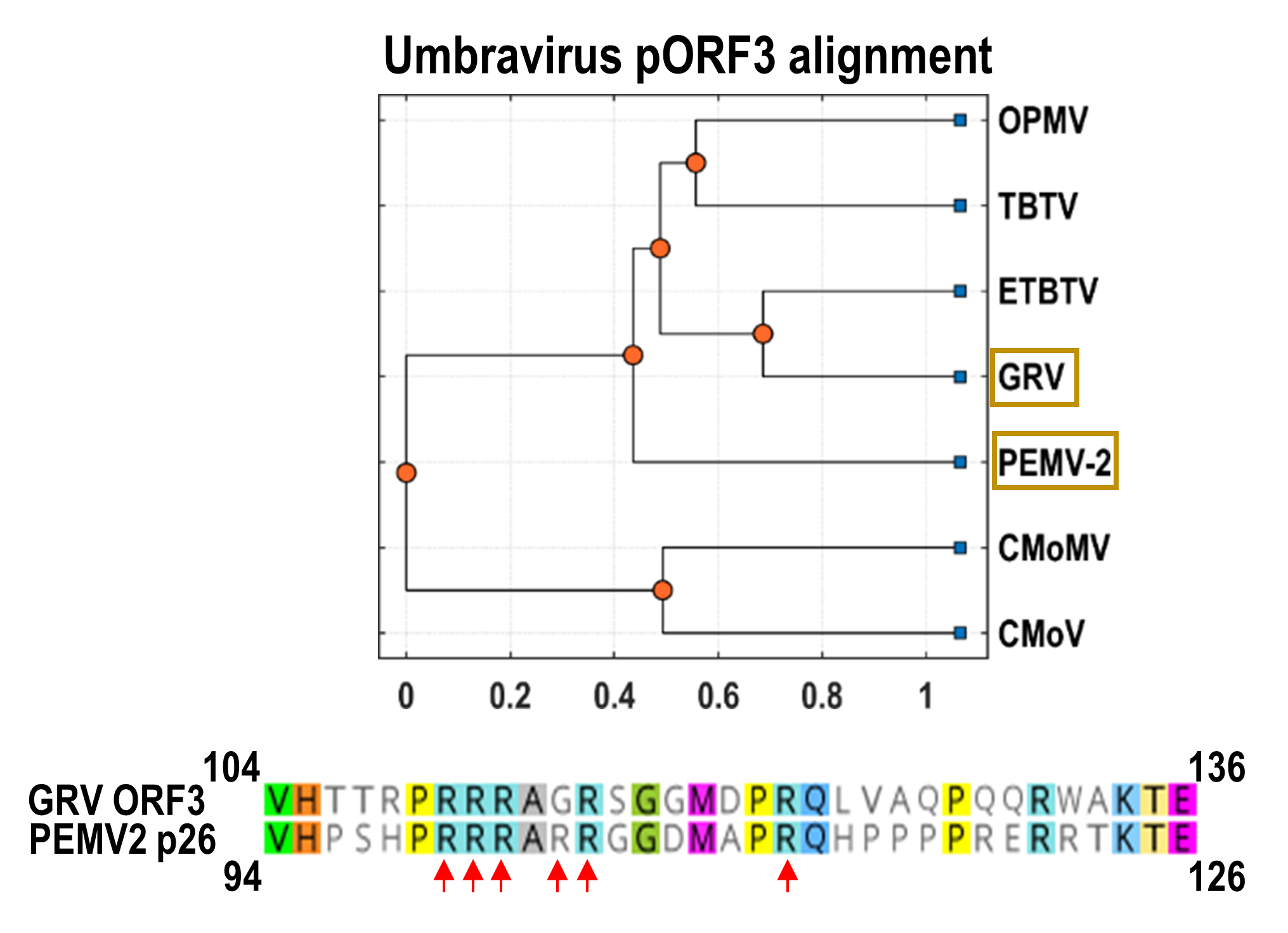

Supplement: FIG S2 [file mBio.00204-20-sf002.tif]

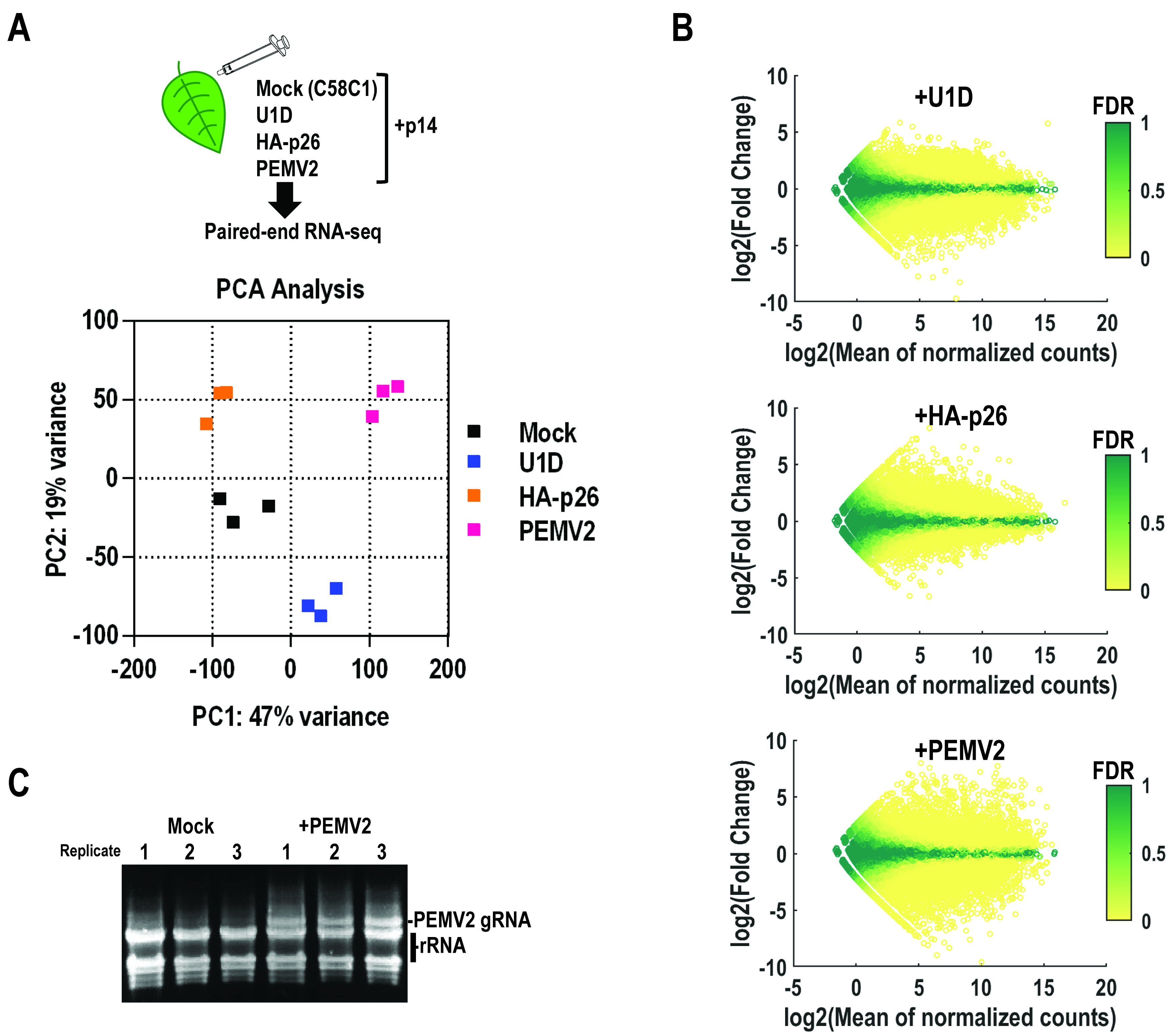

Supplement: FIG S3 [file mBio.00204-20-sf003.tif]

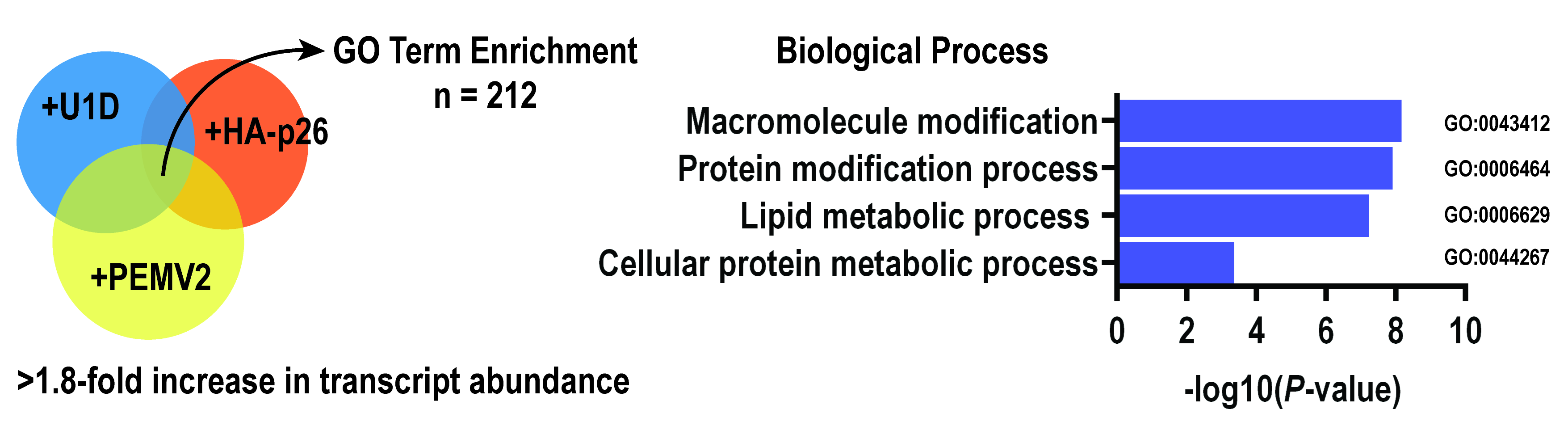

Supplement: FIG S4 [file mBio.00204-20-sf004.tif]

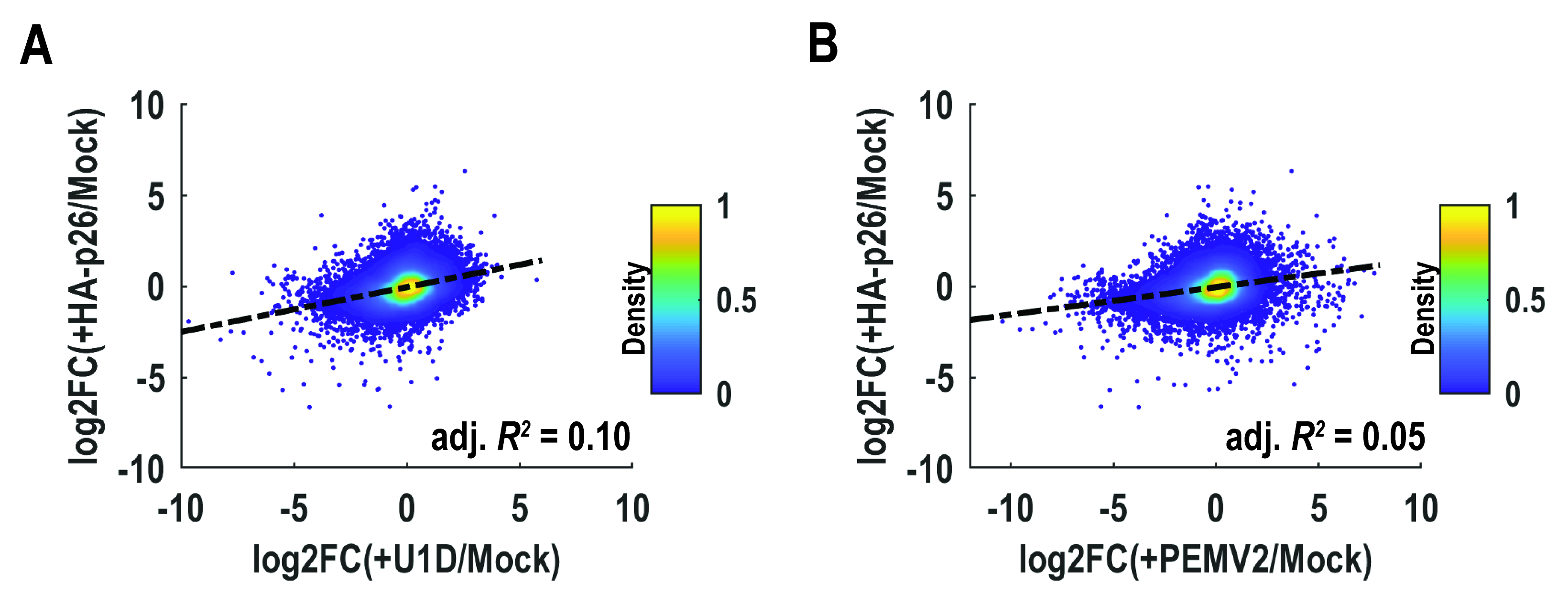

Supplement: FIG S5 [file mBio.00204-20-sf005.tif]

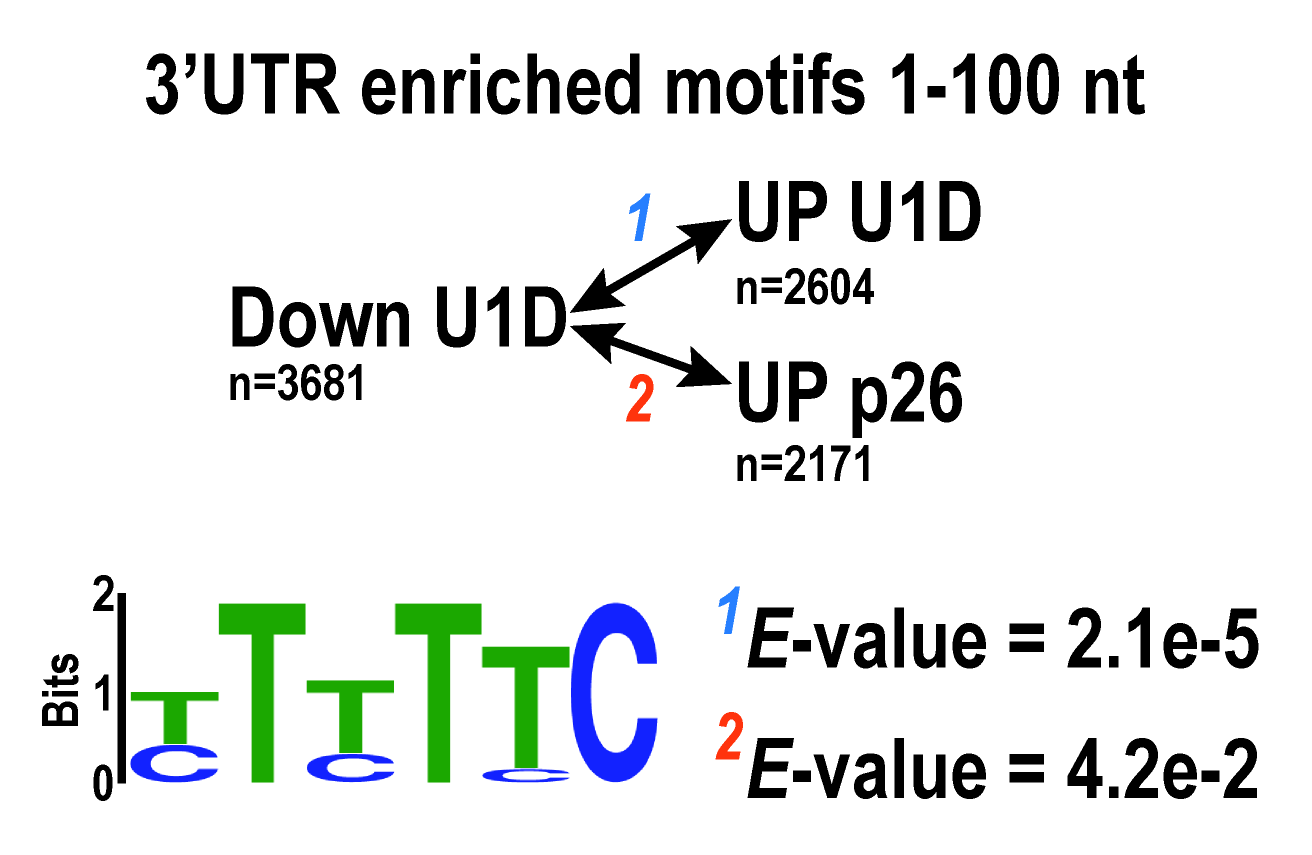

Supplement: FIG S6 [file mBio.00204-20-sf006.tif]
