# Supplementary material for: The Multifunctional Long-Distance Movement Protein of Pea Enation Mosaic Virus 2 Protects Viral and Host Transcripts from Nonsense-Mediated Decay
Source: mBio. 2020 Mar 10;11(2):e00204-20. doi: 10.1128/mBio.00204-20 (PMC7064760; doi:10.1128/mBio.00204-20)
Supplement: TEXT S1 [file mBio.00204-20-s0001.docx]

**Supplemental Information**

**Materials and Methods**

*In vitro p26 expression and translation inhibition assay*. The p26 CDS downstream of the *Tobacco etch virus* (TEV) leader was amplified by PCR (PZP-p26 as template) to introduce a 5′ T7 promoter and 3′ His_6_ tag. RNA transcripts were transcribed and translated in wheat germ extracts (WGE) following the manufacturer’s protocol (Promega). His-tagged p26 was purified by Ni-NTA immobilized metal affinity chromatography (IMAC). Imidazole was removed by sequential desalting and buffer exchange using 10K polyethersulfone (PES) centrifugal membranes (ThermoFisher). Buffer exchange was performed with binding buffer (50 mM Tris-HCl [pH 7.0], 1 mM EDTA, 50 mM NaCl, 1 mM dithiothreitol, and 10% glycerol). Purified p26 or exchange buffer (flow-through) was incubated with FLuc transcripts for 30 minutes prior to *in vitro* translation in WGE. Luciferase activity was measured after 30 minutes of translation using firefly luciferase substrate (Promega).

*Gene ontology (GO) term enrichment analysis*. Transcripts upregulated >1.8-fold with the NMD inhibitor (U1D), HA-p26, and full-length PEMV2 were used for GO term enrichment analysis (n=212). First, GO term annotations were downloaded from the PlantRegMap portal (1). Next, Singular Enrichment Analysis (SEA) using AgriGO v2 (2) was performed to identify biological processes that were enriched for transcripts upregulated by all three conditions.

**References**

1. **Jin J, Tian F, Yang DC, Meng YQ, Kong L, Luo J, Gao G.** 2017. PlantTFDB 4.0: toward a central hub for transcription factors and regulatory interactions in plants. Nucleic Acids Res **45:**D1040-d1045.

2. **Tian T, Liu Y, Yan H, You Q, Yi X, Du Z, Xu W, Su Z.** 2017. agriGO v2.0: a GO analysis toolkit for the agricultural community, 2017 update. Nucleic Acids Res **45:**W122-W129.

3. **Bailey TL, Boden M, Buske FA, Frith M, Grant CE, Clementi L, Ren J, Li WW, Noble WS.** 2009. MEME SUITE: tools for motif discovery and searching. Nucleic Acids Res **37:**W202-208.
